# Efficacy of continuous epidural block in acute herpes zoster: Incidence and predictive factors of postherpetic neuralgia, a retrospective single-center study

**DOI:** 10.1097/MD.0000000000004577

**Published:** 2016-08-12

**Authors:** Yoo Na Kim, Dae Woo Kim, Eung Don Kim

**Affiliations:** a Department of Anesthesiology and Pain Medicine, School of Medicine, The Catholic University of Korea, Daejeon St. Mary's Hospital, Daejeon; b Department of Anesthesiology and Pain Medicine, School of Medicine, The Catholic University of Korea, St. Vincent Hospital, Suwon, Korea.

**Keywords:** continuous epidural, herpes zoster, postherpetic neuralgia, predictive factor

## Abstract

The aim of the present study was to investigate efficacy of continuous epidural block for prevent postherpetic neuralgia (PHN) progression in cases of acute herpes zoster with severe pain and also to identify predictive factors for PHN in such conditions.

We retrospectively analyzed the clinical data of patients with herpes zoster who underwent continuous epidural block between March 2013 and October 2015. Time points were set as 1 month, 3 months, and 6 months after zoster onset. PHN was defined as the presence of pain with NRS ≥3 at certain time points.

The incidence of developing PHN was 38.1%, 27.0%, and 19.0% 1 month, 3 months, and 6 months after zoster onset, respectively. Age and duration of catheterization were predictive factors for PHN at 1 month. Age, duration of catheterization, and NRS at first visit were identified as predictive factors for PHN at 3 months. Presence of diabetes, duration of catheterization, and NRS during catheterization were significant predictive factors for PHN at 6 months.

The incidence of PHN is higher in zoster patients with severe pain that requires continuous epidural block compared to incidence in the general population. Advanced age and severe initial pain intensity were predictive factors of PHN development. Prolonged catheterization resulting from weak response to treatment strongly suggested progression to PHN.

## 1 Introduction

Postherpetic neuralgia (PHN) is a common neuropathic complication of acute herpes zoster that can negatively affect quality of life. The exact pathophysiology of PHN is not yet fully understood. ^[1]^ Reactivation of latent varicella zoster virus (VZV) in sensory ganglia leads to neuronal damage at both central and peripheral levels. This neural injury can result in neuropathic pain processing such as central sensitization. ^[2]^

Advanced age, severe rash, severe pain, and the presence of a prodrome are known risk factors of PHN.[ ^3^ ^4^] To reduce the development of PHN, early administration of an antiviral agent is generally recommended.[ ^5^ ^6^]

Severe pain with acute herpes zoster also is one risk factor of PHN.[ ^3^ ^4^] Nerve blocks can also be used to decrease progression to PHN by halting nociceptive input. ^[7]^ Epidural blocks targeted to the involved dermatome might be helpful for decreasing pain intensity during the acute phase of herpes zoster. However, the efficacy of a single epidural block for preventing PHN is still controversial.[ ^8^ ^9^]

A continuous epidural block theoretically ceases the accumulation of nociceptive input that might contribute to PHN, possibly reducing its development. Although patients who undergo a continuous epidural block are usually concerned about the possibility of PHN, clinical outcomes such as progression to PHN after continuous epidural block have not been investigated before.

In the present study, the incidence of PHN after continuous epidural block was retrospectively evaluated to estimate clinical outcomes of this procedure in acute herpes zoster with severe pain. Predictive factors for PHN after continuous epidural block were also analyzed.

## 2 Method

### 2.1 Study design, setting, and participants

Permission to conduct this study was obtained from the Institutional Ethics Committee of Daejeon St. Mary's Hospital, Republic of Korea (DC16RISI0023). The medical records of patients with herpes zoster who underwent continuous epidural block between March 2013 and October 2015 were retrospectively analyzed.

In our pain clinic, hospital admission is usually recommended in patients with acute herpes zoster with severe symptoms. Epidural catheterization is then performed in cases of intractable pain. If there is no specific renal or hepatic dysfunction, acyclovir 750 mg/day is given intravenously for 7 days.

Catheterizations were conducted to patients with acute herpes zoster affecting the cervical to sacral dermatomes and were not performed to patients with facial involvement. To evaluate efficacy of continuous epidural block for preventing PHN in acute phase of herpes zoster, cases in which it took >1 month to initiate a continuous epidural block were excluded in present analysis.

### 2.2 Procedure

After obtaining written informed consent for the procedure, all patients were positioned prone. An 18-gauge Tuohy needle was introduced into the 2 or 3 below interlaminar space to the target level. The loss of resistance (LOR) technique was used to confirm its presence in the epidural space. Then, a 20-gauge epidural catheter was inserted though the Tuohy needle and positioned at the affected level.

After confirming catheter tip position, continuous infusion of 0.187% ropivacaine (1/4 dilution of 0.75% ropivacaine) with an infusion rate of 1 mL/h was initiated. Ropivacaine concentration and infusion rate were adjusted as the degree of pain reduced. Catheters were removed when patients reported sufficient pain reduction. The duration of catheterization was limited to 2 weeks because of concerns regarding infection. After catheter removal, patients were followed up until pain associated with zoster disappeared. Medical records until remission of zoster pain were analyzed. If zoster pain maintained, the medical record analysis was performed until 6 months after zoster onset.

### 2.3 Data collection

The following data were collected and analyzed: age, sex, body mass index (BMI), whether or not antiviral therapy started within 72 hours after zoster onset, time to initiate continuous epidural block from zoster onset (Time cath), presence of allodynia at first visit (allodynia baseline), numerical rating scale (NRS) at first visit (NRS baseline), NRS just before catheter removal (NRS cath), and NRS 1 month, 3 months, and 6 months after zoster onset. Laboratory findings at first visit were also analyzed, including white blood cell (WBC) count, erythrocyte sedimentation rate (ESR), C-reactive protein (CRP), and VZV immunoglobulin M (VZV IgM) positivity.

The presence of underlying disease was also evaluated and categorized by cardiovascular disease, diabetes, respiratory disease, and hepatobiliary disease.

### 2.4 Definition of PHN

Time points were set at 1 month, 3 months, and 6 months after zoster onset. PHN was defined as the presence of pain with NRS ≥3 at certain time points. Cases with pain intensity of NRS <3 or treatment-terminated cases owing to pain disappearance were defined as non-PHN. Patients were divided into a PHN group (1-month PHN, 3-month PHN, and 6-month PHN) and a non-PHN group at certain time points. The incidences of PHN at each time points after continuous epidural block were evaluated.

### 2.5 Statistical analysis

Data are presented as mean ± standard deviations (SD) for continuous variables.

Repeated measures analysis of variance was used to access changes of pain intensity over time. For univariate analysis, *χ* ^2^ test or Fisher exact test was used for categorical variables, and independent *t* test was used for continuous variables to identify the relevant factors for developing PHN. To determine independent risk factors associated with the development of PHN, binary logistic regression analysis was performed. Variables were screened by examining for multicollinearity (variables were excluded if correlation coefficient *r* > 0.7) and were further selected with a forward stepwise procedure (entry criteria *P* = 0.05, removal criteria *P* = 0.10). All data were analyzed using SPSS version 18.0 (SPSS Inc, Chicago, IL), and *P* values of <0.05 were considered statistically significant.

## 3 Results

**Figure 1:**
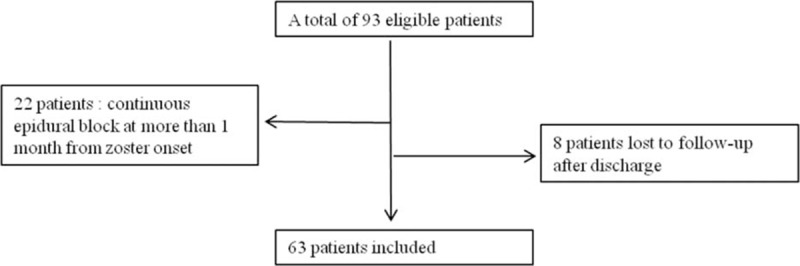
Flow diagram describing patient selection.

We identified 93 patients who underwent epidural catheterization for continuous infusion of local anesthetics associated with herpes zoster. The procedure was performed at >1 month after zoster onset in 22 patients, and clinical follow-ups could not be continued until treatment termination in 8 patients (Fig. 1). Therefore, a total of 63 patients were included in the present retrospective analysis. Demographic data of participants are summarized in Table 1. The incidence of developing PHN was 38.1%, 27.0%, and 19.0% at 1 month, 3 months, and 6 months after zoster onset, respectively (Table 2).

**Table 1:**
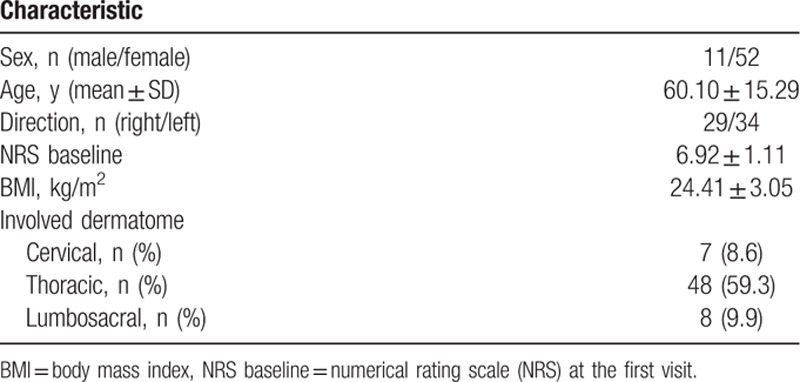
Demographic data of patients.

**Table 2:**
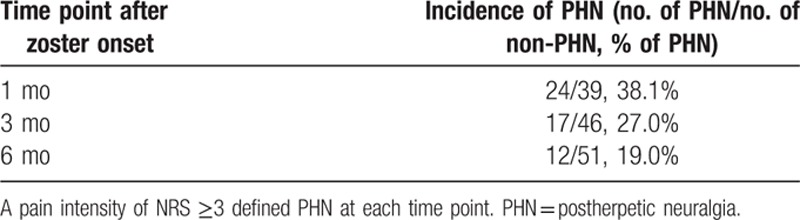
Incidence of PHN at various time points.

**Figure 2:**
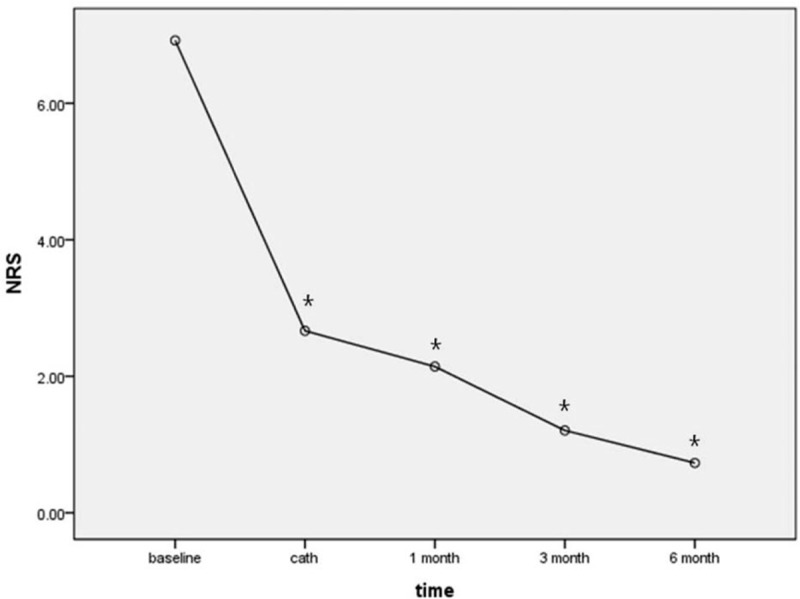
Overall change of NRS over time. NRS was reduced significantly at all time points compared to baseline. NRS = numerical rating scale. ^∗^ *P* < 0.0001 compared to baseline.

**Figure 3:**
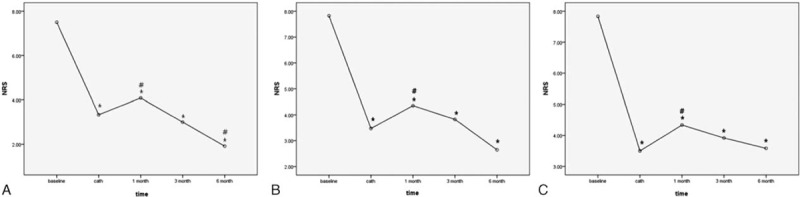
Changes of NRS in PHN groups. (A) 1-month PHN, (B) 3-month PHN, (C) 6-month PHN. Cath = time point just before catheter removal, NRS = numerical rating scale, PHN = postherpetic neuralgia. ^∗^ *P* < 0.05 compared to baseline. #*P* < 0.05 compared to the catheterization period.

The overall NRS of participants significantly reduced with time (Fig. 2). However, in all PHN groups, NRS significantly increased compared to NRS cath after removing the epidural catheter (Fig. 3).

In univariate analysis, age, NRS baseline, and NRS cath were significantly higher in the PHN group at all time points. At 1 month, Time cath was significantly longer in thePHN group (11.71 ± 9.12 vs. 7.74 ± 4.67 days, *P* = 0.026). Durations of catheterization were significantly longer in the PHN group at all time points (1 month: 12.46 ± 3.16 vs.8.64 ± 3.22 days, *P* < 0.0001, 3 months: 12.82 ± 3.34 vs. 9.09 ± 3.23 days, *P* < 0.0001, 6 months: 13.33 ± 3.37 vs. 9.33 ± 3.35 days, *P* < 0.0001). Concentrations of ropivacaine were also significantly higher in PHN group at all time points (1 month: 0.21% ± 0.03% vs.0.19% ± 0.002%, *P* = 0.001, 3 months: 0.22% ± 0.03% vs. 0.19% ± 0.02%, *P* = 0.006, 6 months: 0.21% ± 0.03% vs. 0.19% ± 0.01%, *P* = 0.005). The presence of cardiovascular disease was significantly more frequent in the 3 months PHN group (*P* = 0.047). Diabetes was significantly more frequent in the 3-month PHN and 6-month PHN groups (3-month PHN: *P* = 0.009, 6-month PHN: *P* = 0.009). Among the laboratory findings, only ESR was significantly higher in the 1-month PHN group (*P* = 0.015). Presence of allodynia baseline was significantly related to PHN at all time points (1-month PHN: *P* = 0.0001, 3-month PHN: *P* = 0.0001, 6-month PHN: *P* = 0.002). Because all cases without allodynia baseline did not progress to PHN at all time points, the odds ratio could not be determined from the relationship between presence of allodynia and development of PHN. Therefore, allodynia baseline was an inappropriate variable for logistic regression analysis in this study. This variable was analyzed by univariate analysis using the *χ* ^2^ test (Table 3).

**Table 3:**
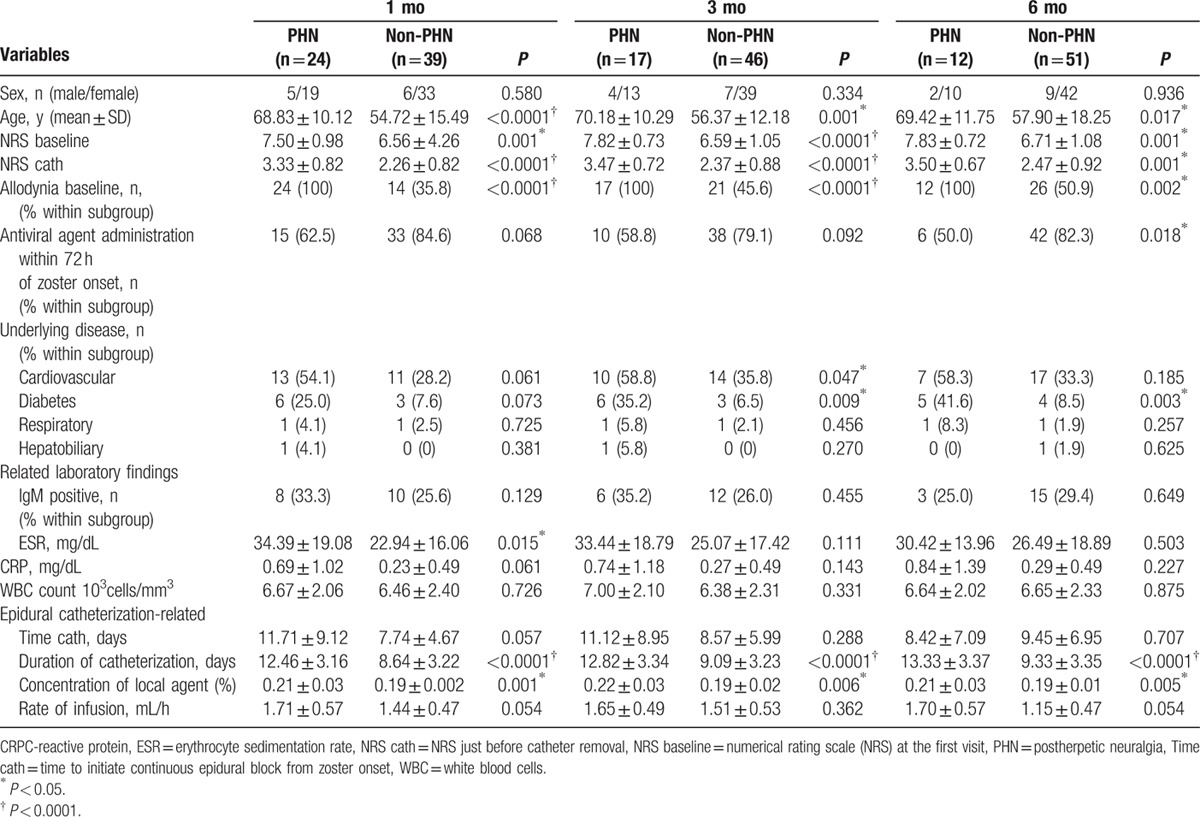
Univariate analysis of PHN vs. non-PHN groups 1 month, 3 months, and 6 months after zoster onset.

Variables found to be significant in univariate analysis and well-known predictable factor of PHN such as antiviral agent starting within 72 hours after the rash onset and presence of underlying disease were included to multivariate logistic regression analysis. Reference variables included absence of cardiovascular disease, absence of diabetes, and initiating antiviral therapy within 72 hours after zoster onset. After forward selection, 4 variables (age, duration of catheterization, NRS cath, ESR), 3 variables (age, duration of catheterization, NRS baseline), and 3 variables (presence of diabetes, duration of catheterization, NRS cath) were identified at time points of 1 month, 3 months, and 6 months, respectively.

Age (OR: 0.872; 95% CI: 0.786–0.969; *P* = 0.011) and duration of catheterization (OR: 0.596; 95% CI: 0.422–0.840; *P* = 0.003) were found to be predictive factors for 1-month PHN. Age (OR: 0.829; 95% CI: 0.737–0.932; *P* = 0.002), duration of catheterization (OR: 0.565; 95% CI: 0.384–0.831; *P* = 0.004), and NRS baseline (OR: 0.187; 95% CI: 0.055–0.633; *P* = 0.007) were identified as predictive factors for 3-month PHN. Presence of diabetes (OR: 0.089; 95% CI: 0.012–0.667; *P* = 0.019), duration of catheterization (OR: 0.684; 95% CI: 0.496–0.948; *P* = 0.023), and NRS cath (OR: 0.289; 95% CI: 0.089–0.935; *P* = 0.039) were significant predictive factors of 6-month PHN (Table 4).

**Table 4:**
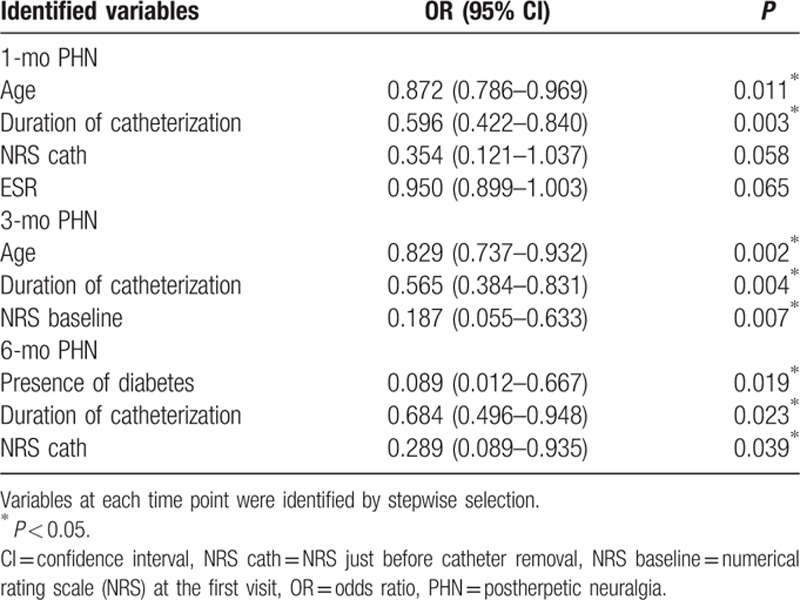
Multivariate logistic regression analysis of independent risk factors associated with the development of PHN.

## 4 Discussion

Continuous afferent nociceptive input caused by the damaged nerve leads to central sensitization, one of the main mechanisms of PHN development. If there is sustained nociceptive input, continuous local infusion by epidural catheterization is theoretically suitable for managing pain.

A significantly shorter time to cessation of zoster pain after continuous epidural block has been reported. ^[10]^ However, that study focused on sensory abnormalities such as allodynia after the procedure. Exact pain intensity, such as NRS through the time course, was not analyzed in that study. Of course, evoked pain such as allodynia is an important clue to diagnosing neuropathic pain. However, underlying sustained pain is also important to neuropathic pain such as PHN. ^[11]^ Therefore, prediction of PHN cannot be determined by the presence of allodynia itself. Moreover, the inclusion criteria of that study were limited the sample to those older than 50 years and cases treated within 10 days of rash onset. The actual incidence of PHN might be higher than the percentage of cases in which allodynia remained. In our clinical experience, sustained pain after continuous epidural block in zoster patients is not a rare phenomenon.

The incidence and predictive factors for PHN in the general population have already been investigated in several studies.[ ^3^ ^4^ ^12^ ^13^ ^14^] To the best of our knowledge, there has been no report regarding the incidence and predictive factors of PHN confined to cases with severe pain or certain procedure such as continuous epidural block. The mean initial NRS of participants in the present study was 6.92 ± 1.11, which could be considered severe.

There is no established consensus regarding the incidence and discriminative time point of PHN and incidences of PHN are widely variable between studies. ^[15]^ However, PHN is generally considered pain that persists for >90 days after the onset of acute zoster rash. ^[16]^ Moreover, discriminative time point of PHN was considered as 90 days in majority of studies. ^[15]^ At 3 months after zoster onset, PHN incidence after continuous epidural block in our study seems to be higher than previously reported PHN incidences of general population ^[15]^ (27.0% vs. range of 2.6%–25%, *P* < 0.0001) and this might be resulted from relatively severe symptom nature in our participants.

Advanced age was significant related with PHN group at all time points by univariate analysis, as in previous studies.[ ^3^ ^4^] Advanced age also was a significant predictive factor for 1-month PHN and 3-month PHN.

In the general population, early initiation of antiviral agent administration[ ^5^ ^6^] and early intervention for pain reduction ^[7]^ are important for resolving zoster pain. Interestingly, starting antiviral therapy within 72 hours after zoster onset was significantly related to only 6-month PHN in univariate analysis. Timing of antiviral therapy was not identified as an entry variable by multivariate logistic regression analysis. Although Time cath was significantly longer in the 1-month PHN group, it was not a meaningful predictive factor for PHN at all time points.

NRS baseline was significantly higher in PHN groups at all time points, in agreement with recent systemic review. ^[15]^ NRS cath was also higher in all PHN groups. NRS baseline and NRS cath were significant predictive factors of 3-month PHN and 6-month PHN, respectively. Therefore, both initial pain intensity and degree of pain reduction during catheterization are useful predictive factors for PHN after continuous epidural block.

Duration of catheterization was also significantly longer in PHN groups at all time points on univariate analysis. Distinct from NRS baseline and NRS cath, duration of catheterization was a significant predictive factor of PHN at all time points. Requiring more time to decrease the intensity of zoster pain implies more severe nerve damage caused by VZV infection. The possibility of increasing pain after halting local agent infusion might be higher in herpes zoster patients with severe nerve damage. Therefore, duration of catheterization could be a strong independent risk factor related to the development of PHN in cases of acute zoster with catheterization.

One study reported an increased risk of PHN with allodynia. ^[17]^ However, other studies reported no effect of allodynia on PHN development.[ ^13^ ^18^] In this study, the presence of allodynia baseline was significantly associated with PHN at all time points. Allodynia is a neuropathic symptom associated with dorsal horn reorganization, such as sprouting of Aβ fibers. ^[11]^ If symptoms suggesting central sensitization exist at an earlier stage, the risk of PHN may increase.

Among laboratory findings that reflect inflammation, such as ESR, CRP, and WBC count, only ESR was significantly high in the 1-month PHN group. This result is discordant with previous findings, ^[19]^ and it might be attributed to the relatively severe nature of symptoms in the present study.

Diabetes has been reported as a risk factor of PHN in previous studies.[ ^15^ ^18^] In the present study, presence of diabetes was also identified as a risk factor of 6-month PHN. However, the OR of this variable was relatively low.

The thoracic level is the most commonly affected area, and patients with zoster who undergo continuous epidural block are often elderly. Therefore, excessive hemodynamic changes owing to intervention should be avoided. Ectopic discharge from injured nerves is suppressed by local agents of lower concentrations than those required to block normal nerve conduction and this susceptibility of damaged nerve reported in previous studies.[ ^20^ ^21^] To minimize such hemodynamic changes, we initiated continuous epidural block with a lower concentration of local agent using 0.187% ropivacaine. The concentration was adjusted depending on the degree of pain relief. As a weaker response to treatment was identified as a predictive factor of PHN in the present study, we believe that an application of higher concentrations and infusion rates of local agent than present setting is needed to improve the clinical outcomes of continuous epidural block in herpes zoster.

A relatively small sample size and data from a single center are limitations of the present study. To further investigate the efficacy of continuous epidural block in zoster patients with severe pain, a control group without intervention is needed. However, such a study design is difficult to employ for ethical reasons. The present retrospective analysis provides indirect data to estimate the efficacy of continuous epidural block in severe zoster cases. Unfortunately, the presence of prodromal symptoms and rash severity were not included to this analysis because of insufficient medical records.

In conclusion, the incidence of PHN is higher than that of the general population in zoster patients with severe pain that requires continuous epidural block. Advanced age and high initial pain intensity were meaningful predictive factors for the development of PHN, as shown in previous studies. Prolonged catheterization resulting from weak response to treatment strongly suggested progression to PHN. Active efforts to decrease pain intensity during catheterization with various catheterization settings are important to minimize the development of PHN. An additional procedure such as pulsed radiofrequency on affected nerve root also can be applied in cases of prolonged catheterization by weak response.

Further studies with a larger sample size and multicenter data with various epidural infusion settings are needed to improve the efficacy of this procedure in herpes zoster.
